# Porcine epidemic diarrhea virus activates PERK-ROS axis to benefit its replication in Vero E6 cells

**DOI:** 10.1186/s13567-023-01139-z

**Published:** 2023-02-03

**Authors:** Yingshan Zhou, Yuxin Zhang, Wanyu Dong, Shiqi Gan, Jing Du, Xingdong Zhou, Weihuan Fang, Xiaodu Wang, Houhui Song

**Affiliations:** Key Laboratory of Applied Technology On Green-Eco-Healthy Animal Husbandry of Zhejiang Province, Zhejiang Provincial Engineering Research Center for Animal Health Diagnostics & Advanced Technology, Zhejiang International Science and Technology Cooperation Base for Veterinary Medicine and Health Management, China-Australia Joint Laboratory for Animal Health Big Data Analytics, College of Animal Science and Technology & College of Veterinary Medicine of Zhejiang A&F University, 666 Wusu Street, Lin’an District, Hangzhou, 311300 Zhejiang China

**Keywords:** Porcine epidemic diarrhea virus, endoplasmic reticulum stress, oxidative stress, virus replication

## Abstract

Of the three branches of unfolded protein response (UPR) that were reportedly activated by porcine epidemic diarrhea virus (PEDV), PERK is recently shown to act as an upstream regulator of oxidative response of the cells. However, it remains unknown if and how PERK activation during PEDV infection would result in oxidative stress, and whether activation of PERK and its downstream molecules affect PEDV replication. Here, we demonstrate that infection with the PEDV strain YJH/2015 triggered UPR in Vero E6 cells by activating the PERK/eIF2α pathway and led to significant increase in the expression of proapoptotic protein C/EBP homologous protein (CHOP) and ER oxidoreductase 1 alpha (ERO1α). Inhibition of PERK by short hairpin RNA (shRNA) or GSK2606414 and knockdown of CHOP by small interfering RNA reduced expression of ERO1α and generation of ROS in PEDV-infected cells. Inhibition of ERO1α by shRNA or EN460 decreased PEDV-induced ROS generation. Genetic or pharmacological inhibition of each component of PERK, CHOP, ERO1α, and ROS led to significant suppression of PEDV replication. Collectively, our study provides the first evidence that PEDV manipulates endoplasmic reticulum to perturb its redox homeostasis via the PERK-CHOP-ERO1α-ROS axis in favor of its replication.

## Introduction

Porcine epidemic diarrhea virus (PEDV), a member of the genus *Alphacoronavirus* in the family *Coronaviridae* of the order *Nidovirales*, is the causative agent of porcine epidemic diarrhea (PED), which is characterized by dehydration, vomiting, diarrhea, and high mortality in neonatal piglets [1]. PEDV is an enveloped, single-stranded, non-segmented, positive-sense RNA virus. Its genome is approximately 28 kb in size that encodes four structural proteins, namely, spike (S), envelope (E), membrane (M) and nucleocapsid (N) proteins, sixteen nonstructural proteins (nsp1-nsp16), and an accessory protein ORF3 [2].

During coronavirus replication, viral proteins induce the formation of endoplasmic reticulum (ER)-derived double-membrane vesicles for RNA synthesis, and viral structural proteins assemble the virions at the ER-Golgi intermediate compartment [3]. Association and intense utilization of the ER during viral replication may induce ER stress and unfolded protein response (UPR), a signal transduction cascade that acts to reprogram gene transcription, modulate translation and membrane biosynthesis to relieve the load of unfolded or misfolded proteins and restore protein homeostasis [4]. Accumulating evidence from recent studies has shown that induction of ER stress and UPR may constitute a major aspect of coronavirus-host interaction [5]. Activation of the three branches (i.e., PERK, IRE1 and ATF6) of UPR modulates a wide variety of signaling pathways, such as mitogen-activated protein kinase activation, autophagy, apoptosis, and innate immune responses. ER stress and UPR may, therefore, contribute significantly to viral replication and pathogenesis during coronavirus infection [6].

The endoplasmic reticulum maintains redox conditions that are optimal for the essential process of disulfide-bond formation in nascent proteins of the secretory pathway [7]. Protein folding and generation of reactive oxygen species (ROS) as a byproduct of protein oxidation in the ER are closely linked events [8]. ROS-dependent oxidative stress during ER stress is induced by the C/EBP homologous protein (CHOP)-mediated transcriptional upregulation of ER oxidoreductase 1 alpha (ERO1α), whose activity serves an efficient oxidative protein folding process and produces H_2_O_2_ [9].

Activation of ER stress by microbial infections has been widely observed [10]. However, with few exceptions, it remains unknown how this response is shaped in an infectious agent-specific manner and whether these responses are beneficial or detrimental to viral replication. PEDV was recently reported to induce ER stress and UPR in intestinal epithelial cells of weaned pigs [11] and in Vero E6 cells [12]. Several PEDV viral proteins, such as E protein [13], N protein [14], and ORF3 [15] were also reported to induce ER stress. However, the molecular mechanisms linking ER stress and oxidative stress during PEDV infection are poorly understood. Here, we show that PEDV infection activated the PERK branch of UPR, which in turn upregulated the expression of downstream molecules CHOP and ERO1α, resulting in increased generation of ROS. Downregulation of PERK or inhibition of each of its downstream molecules suppressed PEDV replication.

## Materials and methods

### Cells and virus

African green monkey epithelial cells (Vero E6, ATCC CRL-1586) and human embryonic kidney cells (HEK293T) were maintained at 37 ℃ and 5% CO_2_ in Dulbecco’s modified Eagle’s medium (DMEM, Thermo Fisher Scientific, Marina, CA, USA) supplemented with 10% (vol/vol) heat inactivated newborn calf serum (Gibco, Grand Island, NY, USA), 1% L-glutamine, 1% non-essential amino acids, 100 U/mL penicillin G, and 100 μg/mL streptomycin. PEDV strain YJH/2015 (GenBank accession number MT646162.1) was isolated from a naturally infected piglet and propagated in Vero E6 cells.

### Virus infection and chemical treatments

Vero E6 cells were infected with PEDV at multiplicity of infection (MOI) of 0.001. After adsorption at 37 °C for 2 h, the supernatant was removed. The cell monolayers were washed with sterile phosphate buffered saline (PBS) at pH 7.4 to remove unbound viruses and then incubated in DMEM supplemented with 0.2% trypsin (0.25%; Gibco) at 37 °C for indicated time points.

Vero E6 cells were infected as described above and cultured in fresh medium in the absence or presence of the following chemical: 5 μM PERK inhibitor GSK2606414 (MedChemExpress, Jersey, NJ, USA), 5 μM ERO1α inhibitor EN460 (MedChemExpress), 10 mM antioxidant *N*-acetyl-L-cysteine (NAC) (Sigma-Aldrich, St. Louis, MO, USA), or the solvent control (dimethylsulfoxide, DMSO). The cells were harvested at 36 h post-infection (hpi) for Western blotting, and the supernatants were harvested for viral titration.

### Establishment of *perk*- or *ero1α*-knockdown Vero E6 cells

Short hairpin RNAs (shRNAs) targeting monkey *perk* (5′-GGACATAGTGATAAAGGTTTC-3′) and monkey *ero1α* (5′-GCTGTCAAACCATGTCAATCT-3′) as well as the scrambled control (shNC) sequence (5′-TTCTCCGAACGTGTCACGT-3′) were synthesized and annealed into linear pLKO.1 lentiviral vector. Lentivirus production was performed as described previously [16]. Briefly, HEK293T cells were cotransfected with recombinant lentiviral vector and the helper plasmids psPAX2 and pMD2.G, using jetPRIME reagent (Polyplus, Illkirch, France). The culture supernatant samples containing viral particles were collected and used to transduce the Vero E6 cells to generate *perk*-knockdown (shPERK) or *ero1α*-knockdown (shERO1α) cells in the presence of 5 μg/mL polybrene (Sigma-Aldrich) for 72 h and subjected to puromycin selection (5 μg/mL, Sigma-Aldrich).

### RNA interference

Small interfering RNA (siRNA) duplexes against monkey *chop* and scrambled siRNA as control were synthesized by Genepharma (Shanghai, China). The sense strand of siRNA for *chop* was 5'- GGAGAAAGAACAGGAGAAUTT -3'. siRNA was delivered into Vero E6 cells by transfection with jetPRIME reagent (Polyplus) according to manufacturer’s instructions.

### Western blotting

Vero E6 cells infected with PEDV and/or receiving various treatments were lysed in lysis buffer containing cOmplete™ protease inhibitor cocktail (Roche Applied Science, IN, USA) after infection or chemical treatments for indicated time points. The lysates were harvested and protein concentrations were quantified by using a bicinchoninic acid assay kit. Equal amounts of proteins of the cell lysate samples were separated by sodium dodecyl sulfate–polyacrylamide gel electrophoresis (SDS-PAGE) and transferred onto polyvinylidene difluoride membrane (Millipore, Billerica, MA, USA). The membranes were blocked for 1 h in Tris-buffered saline containing 0.05% Tween 20 and 5% nonfat milk and then incubated at 4 °C overnight with the following primary antibodies: mouse monoclonal anti-PEDV-N protein IgG (produced in our laboratory), rabbit monoclonal antibodies to GRP78 (Abcam, Cambridge, UK), PERK (Cell Signaling Technology (CST), Massachusetts, USA), eIF2α (CST), ERO1α (CST), CHOP (Huabio, Hangzhou, China), p-PERK (Abmart, Shanghai, China), p-eIF2α (Abcam), and mouse monoclonal antibody to β-actin (MultiSciences, Hangzhou, China). Blots were washed and incubated for 1 h with goat anti-rabbit or anti-mouse horseradish peroxidase-labeled antibodies (KPL, Gaithersburg, MD, USA). The blots were revealed using ECL Plus Western Blotting Substrate (Thermo Fisher Scientific). Images were captured by the Gel 3100 Chemiluminescent and Fluorescent Imaging System (Sagecreation, Beijing, China). The band density was quantified using Image J Software (version 1.51j8, National Institutes of Health (NIH), Bethesda, MD, USA) with normalization to the β-actin signal.

### Measurement of cytosolic ROS

Cytosolic ROS in infected or chemical treated Vero E6 cells were measured after treatment with 10 μM 2ʹ,7ʹ-dichlorodihydrofluorescein diacetate (DCFH-DA) (Solarbio, Beijing, China) for 30 min at 37 °C in the dark. The cells were washed twice with PBS, harvested after 0.25% trypsin treatment for centrifugal precipitation (3 min at 500 × *g*). The cell pellets were resuspended in PBS at a concentration of 10^6^ cells/mL for flow cytometric analysis on the BD flow cytometer (BD Accuri™, NJ, USA). Data analysis was performed using the FlowJo software (version 10, TreeStar).

### Quantification of viral RNA by real-time RT-PCR

Total RNA was extracted using TRIzol Reagent (Ambion, USA) and used as the template for cDNA synthesis using HiScript III All-in-one RT SuperMix (Vazyme Biotech, Nanjing, China). The synthesized cDNA was analyzed by real-time RT-PCR using Taq Pro Universal SYBR qPCR Master Mix (Vazyme Biotech) in a qPCR machine (Aglient, Mx3000P, USA). The cycling profile was as 95 °C for 30 s; 40 cycles of 95 °C for 10 s, 60 °C for 30 s; and 1 cycle of 95 °C for 15 s, 60 °C for 60 s, 95 °C for 15 s. The recombinant plasmid containing the PEDV S gene, which was run for qPCR under the same condition after serial dilutions, was used to construct a standard curve for interpolation of viral RNA quantity. Results were expressed as the mean of the logarithmic viral RNA copies per μL. Primers were: PEDV-F: 5′-GTTCTTTTCAAAATTTAATGTTCAGGC-3′ and PEDV-R: 5′-GAAATGCCAATCTCAAAGCC-3′.

### TCID_50_

The supernatant samples containing virions, collected as described above, were clarified by centrifugation at 8000 × *g* for 10 min prior to titration. Fifty percent tissue culture infective dose (TCID_50_) assays were performed on Vero E6 cells according to the method of Reed and Muench. Briefly, cell monolayers (10^4^ cells per well) in 96-well tissue culture plates (Corning, NY, USA) were inoculated with 100 μL tenfold serial dilutions of each virus stock and incubated for 4 days prior to observation of the cytopathic effect.

### Statistical analysis

Data are expressed as means ± standard deviations from three independent experiments and analyzed by using the Student’s *t*-tests. Differences were considered significant with *P* values < 0.05 (*) and highly significant with *P* values < 0.01 (**) or < 0.001 (***).

## Results

### PEDV infection upregulated CHOP and ERO1α expression and induced cytosolic ROS

**Figure 1:**
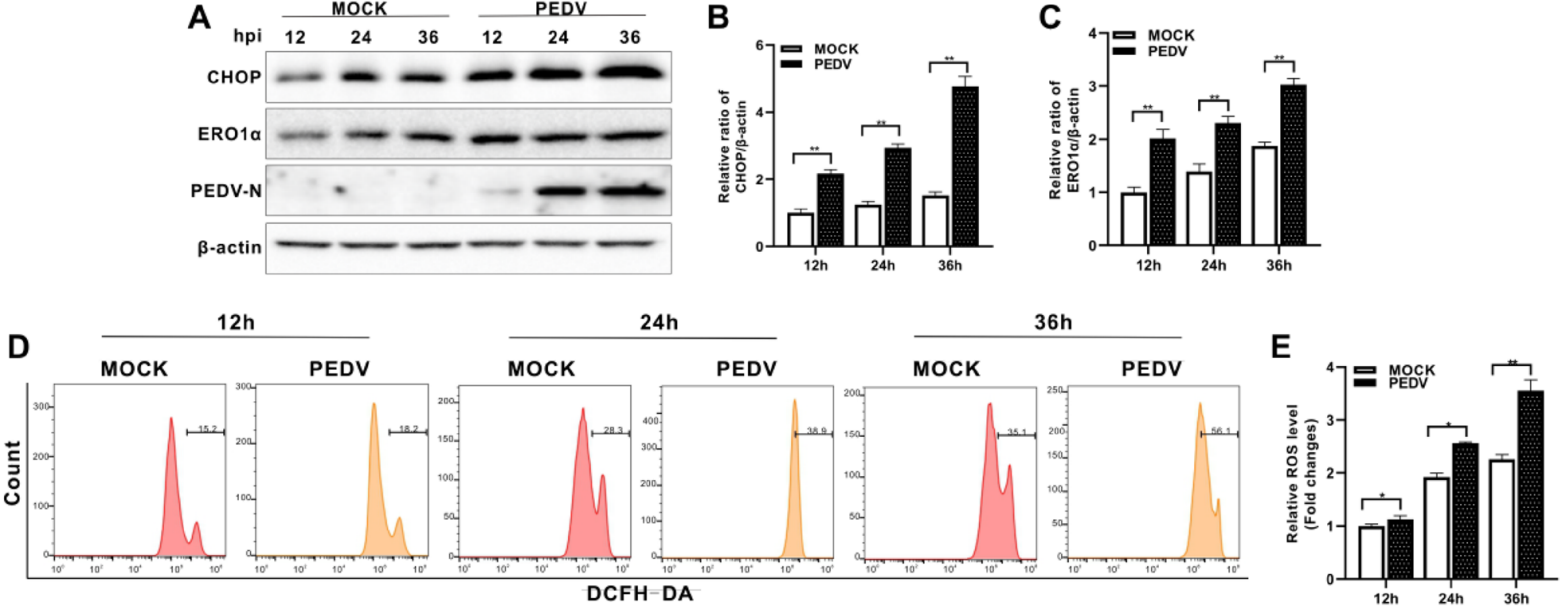
**PEDV infection increased the levels of CHOP and ERO1α expression and cytosolic ROS.** Vero E6 cells were infected with PEDV (MOI = 0.001) or mock infected for indicated time points. **A** Western blotting of CHOP and ERO1α. β-actin was used as loading control. **B** Ratios of CHOP to β-actin. **C** Ratios of ERO1α to β-actin. **D** Cells were measured for changes in ROS level by flow cytometry after incubation with 10 μM DCFH-DA for 30 min at 37 °C. **E** The changes in cytosolic ROS levels were evaluated according to the mean value of DCFH-DA fluorescence intensity and expressed as fold changes between mock- and PEDV-infected cells. The bar charts in **B, C,** and **E** represent mean ± SD of three independent experiments. *, *P* < 0.05; **, *P* < 0.01.

Accumulation of misfolded proteins causes ROS generation from the oxidative folding machineries in the ER. Defective disulfide bond formation depletes glutathione in the ER and produces oxygen radicals via ERO1α [17]. Prolonged PERK/eIF2α pathway activation is known to closely intertwined with oxidative stress. In a separate experiment on the induction of UPR by our PEDV strain YJH/2015, we found that this strain did activate PERK/eIF2α (data not shown) which were similar to the findings reported by Sun et al. [12]. An important mechanism linking PERK with ROS generation involves induction of apoptogenic transcription factor CHOP, which upregulates ERO1α through binding to its promotor [18]. Thus, we investigated CHOP expression in PEDV-infected Vero E6 cells by Western blotting. PEDV infection markedly upregulated CHOP expression from 12 to 36 hpi (Figures 1A and B). ERO1α was also significantly upregulated in parallel to increased CHOP expression (Figures 1A and C). By flow cytometric analysis, we found that PEDV infection induced elevation of cytosolic ROS (Figures 1D and E).

### Inhibition of PERK mitigated PEDV-induced elevation of ROS and suppressed virus replication

**Figure 2:**
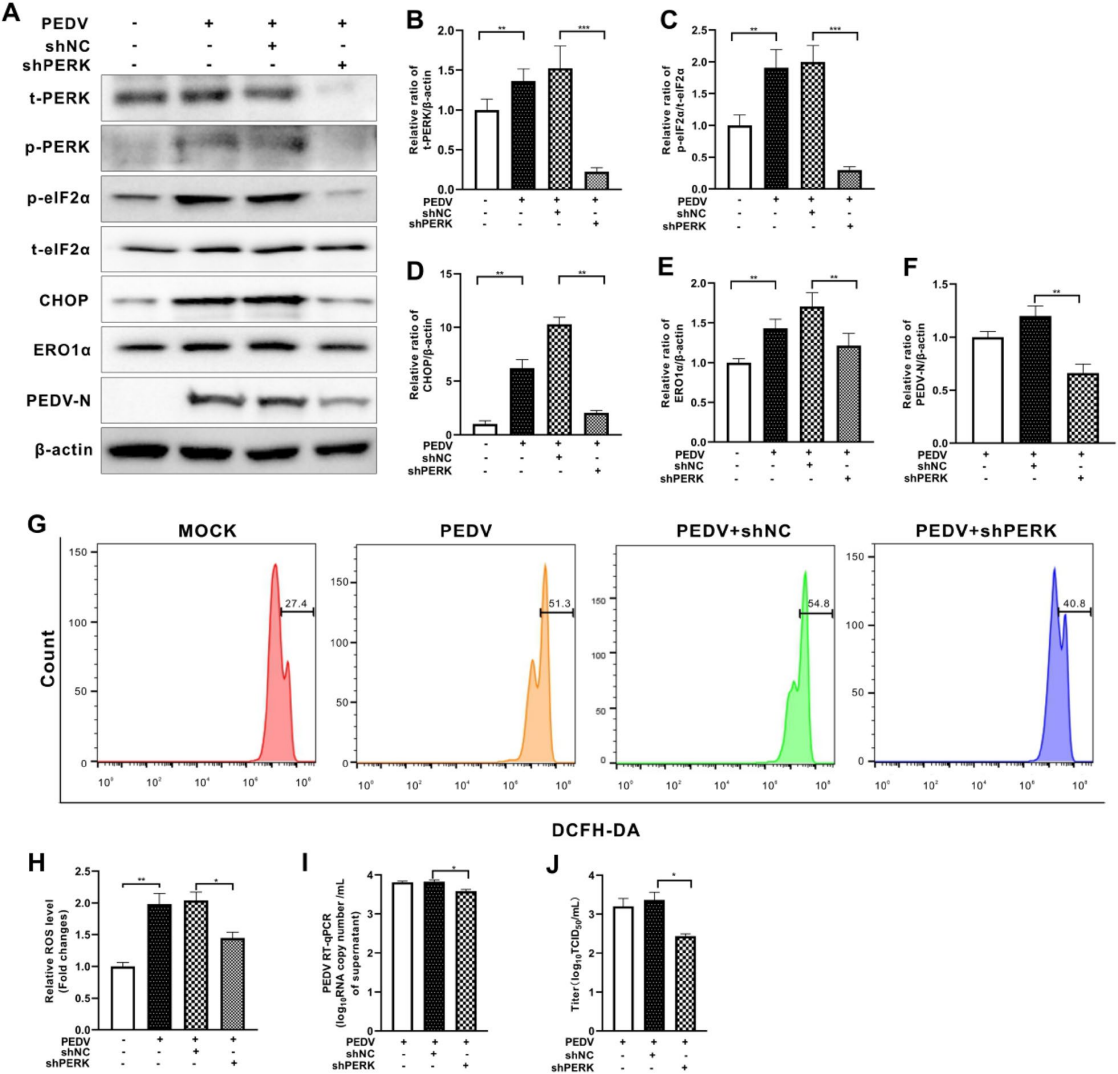
**Inhibition of PERK reduced cellular ROS levels and viral replication in PEDV-infected Vero E6 cells.** Both the PERK-silenced (shPERK) Vero E6 cells and control cells (shNC) were infected with PEDV (MOI = 0.001) for 36 h. **A** The effects of *perk* knockdown on eIF2α, CHOP, ERO1α, and PEDV N expression were shown by Western blotting. β-actin was used as loading control. **B** Ratios of PERK to β-actin. **C** Ratios of p-eIF2α to t-eIF2α. **D** Ratios of CHOP to β-actin. **E** Ratios of ERO1α to β-actin. **F** Ratios of N to β-actin. **G** Cells collected at 36 hpi were measured for changes in cytosolic ROS level by flow cytometry after incubation with 10 μM DCFH-DA for 30 min at 37 °C. **H** The changes in cytosolic ROS levels were evaluated according to the mean value of DCFH-DA fluorescence intensity and expressed as fold changes between mock- and treated-cells. The effects of PERK inhibition by shRNA on viral replication, shown as viral RNA copy numbers measured by RT-qPCR (**I**) and virus titer (**J**). The bar charts in **B** to **F**, **H** to **J** represent mean ± SD of three independent experiments. *, *P* < 0.05; **, *P* < 0.01; ***, *P* < 0.001.

**Figure 3:**
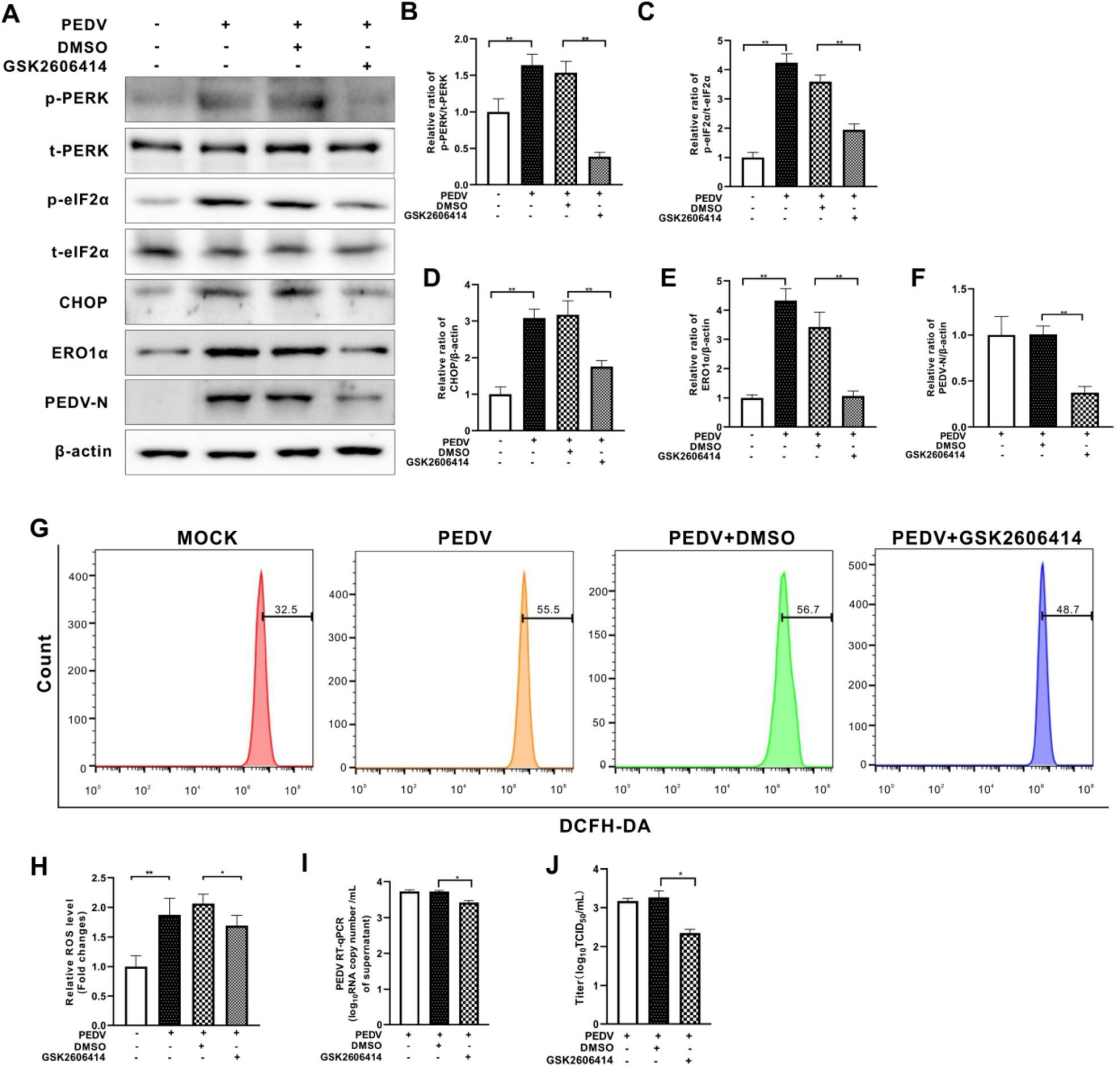
**Inhibition of PERK by GSK2606414 reduced cellular ROS levels and viral replication in PEDV-infected Vero E6 cells.** Vero E6 cells were infected with PEDV (MOI = 0.001) with or without GSK2606414 (5 μM) for 36 h, dimethylsulfoxide (DMSO) was used as solvent control. **A** The effects of PERK inhibition on eIF2α phosphorylation, CHOP, ERO1α, and PEDV N expression were shown by Western blotting with β-actin used as loading control. **B** Ratios of p-PERK to t-PERK. **C** Ratios of p-eIF2α to t-eIF2α. **D** Ratios of CHOP to β-actin. **E** Ratios of ERO1α to β-actin. **F** Ratios of N to β-actin. **G** Cells collected at 36 hpi were measured for changes in cytosolic ROS level by flow cytometry after incubation with 10 μM DCFH-DA for 30 min at 37 °C. **H** The changes in cytosolic ROS levels were evaluated according to the mean value of DCFH-DA fluorescence intensity and expressed as fold changes between mock- and treated-cells. The effects of PERK inhibition by GSK2606414 on viral replication, shown as viral RNA copy numbers measured by RT-qPCR (**I**) and virus titer (**J**). The bar charts in **B** to **F**, **H** to **J** represent mean ± SD of three independent experiments. *, *P* < 0.05; **, *P* < 0.01.

To reveal the role of PERK in ROS generation and PEDV replication, we initially downregulated PERK expression by short hairpin RNA (shRNA) (Figures 2A and B). We found that PERK knockdown reduced eIF2α phosphorylation induced by PEDV infection (Figures 2A and C). The expression of CHOP and ERO1α was also significantly reduced (Figures 2A, D and E). Meanwhile, viral replication was also restricted, as revealed by reduced protein levels observed for the viral nucleocapsid (N) protein (a major coronavirus structural protein) (Figures 2A and F), viral RNA copy numbers and virus titer (Figures 2I and J). We further treated cells with the PERK inhibitor GSK2606414. This compound suppressed PERK autophosphorylation and eIF2α phosphorylation, decreased the expression of CHOP and ERO1α, and inhibited PEDV replication (Figure 3). Inhibition of PERK either by shRNA or by GSK2606414 could counteract elevation of ROS levels induced by PEDV infection (Figures 2G, H, 3G, H). These data clearly show that ERO1α expression, increased ROS production, and viral replication are closely linked with PERK activation by PEDV infection.

### CHOP knockdown mitigated PEDV-induced elevation of ROS and virus replication

**Figure 4:**
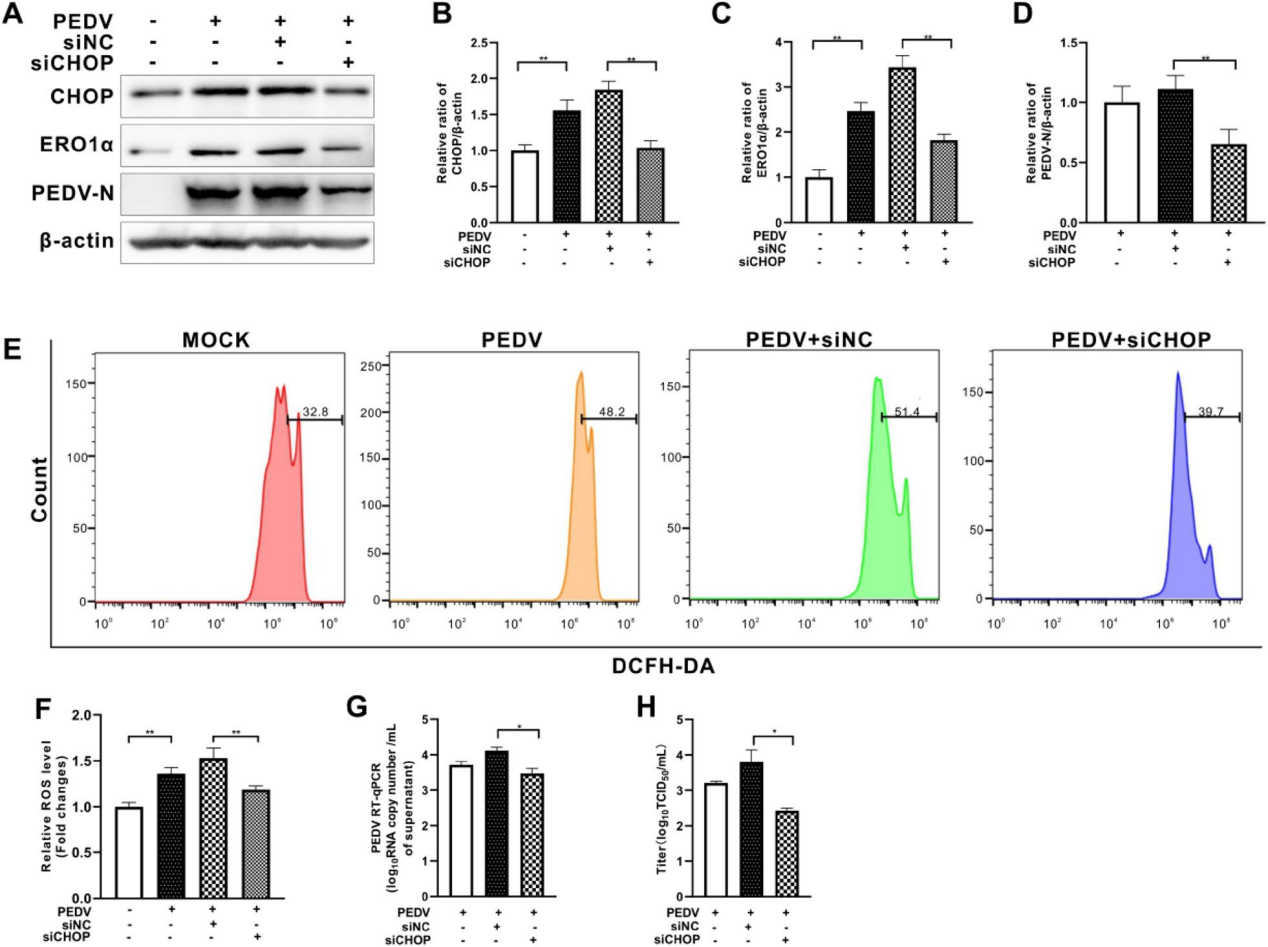
**Small interfering RNA-mediated silence of chop gene reduced cellular ROS levels and viral replication in PEDV-infected Vero E6 cells.** Vero E6 cells were first transfected with *chop*-specific siRNA (siCHOP) or with scrambled RNA as control (siNC). After 6 h of transfection, the cells were infected with PEDV (MOI = 0.001) for 36 h. **A** The effects of CHOP inhibition on ERO1α, and PEDV N expression were shown by Western blotting with β-actin used as loading control. **B** Ratios of CHOP to β-actin. **C** Ratios of ERO1α to β-actin. **D** Ratios of N to β-actin. **E** Cells collected at 36 hpi were measured for changes in cytosolic ROS level by flow cytometry after incubation with 10 μM DCFH-DA for 30 min at 37 °C. **F** The changes in cytosolic ROS levels were evaluated according to the mean value of DCFH-DA fluorescence intensity and expressed as fold changes between mock- and treated-cells. The effects of ERO1α inhibition by shRNA on viral replication, shown as viral RNA copy numbers measured by RT-qPCR (**G**) and virus titer (**H**). The bar charts in **B** to **D**, **F** to **H** represent mean ± SD of three independent experiments. *, *P* < 0.05; **, *P* < 0.01.

We next investigated if CHOP knockdown would have effects on ROS generation and viral replication in PEDV-infected cells. The knockdown efficiency was verified by Western blotting (Figures 4A, B). We found that *chop*-silencing markedly diminished ERO1α protein expression and cytosolic ROS induced by PEDV infection (Figures 4A, C, E, and F). Significant suppression of viral replication by *chop*-silencing was also demonstrated as shown by reduced levels of viral N expression, genomic RNA copy numbers and virus titer (Figures 4A, D, G, H).

### Inhibition of ERO1α relieved PEDV-induced elevation of ROS and virus replication

**Figure 5:**
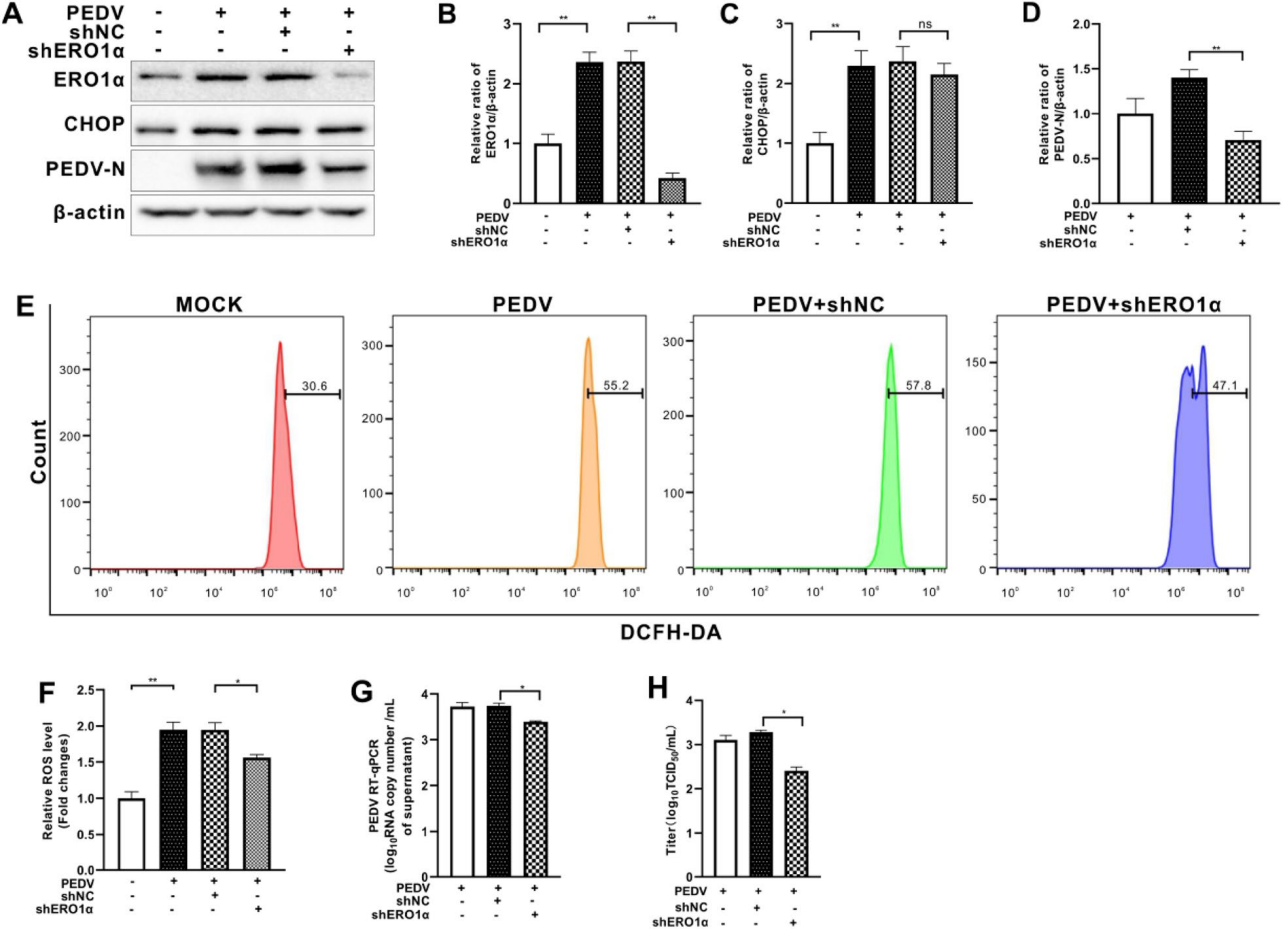
**Silencing of the ero1α gene reduced cytosolic ROS and viral replication in PEDV-infected Vero E6 cells.** Both the ERO1α-silencing (shERO1α) Vero E6 cells and control cells (shNC) were infected with PEDV (MOI = 0.001) for 36 h. **A** The effects of *ero1α* knockdown on CHOP, ERO1α, and PEDV N expression were shown by Western blotting with β-actin used as loading control. **B** Ratios of ERO1α to β-actin. **C** Ratios of CHOP to β-actin. **D** Ratios of N to β-actin. **E** Cells collected at 36 hpi were measured for changes in cytosolic ROS level by flow cytometry after incubation with 10 μM DCFH-DA for 30 min at 37 °C. **F** The changes in cytosolic ROS levels were evaluated according to the mean value of DCFH-DA fluorescence intensity and expressed as fold changes between mock- and treated-cells. The effects of ERO1α inhibition by shRNA on viral replication, shown as viral RNA copy numbers measured by RT-qPCR (**G**) and virus titer (**H**). The bar charts in **B** to **D**, **F** to **H** represent mean ± SD of three independent experiments. ns, not significant; *, *P* < 0.05; **, *P* < 0.01.

**Figure 6:**
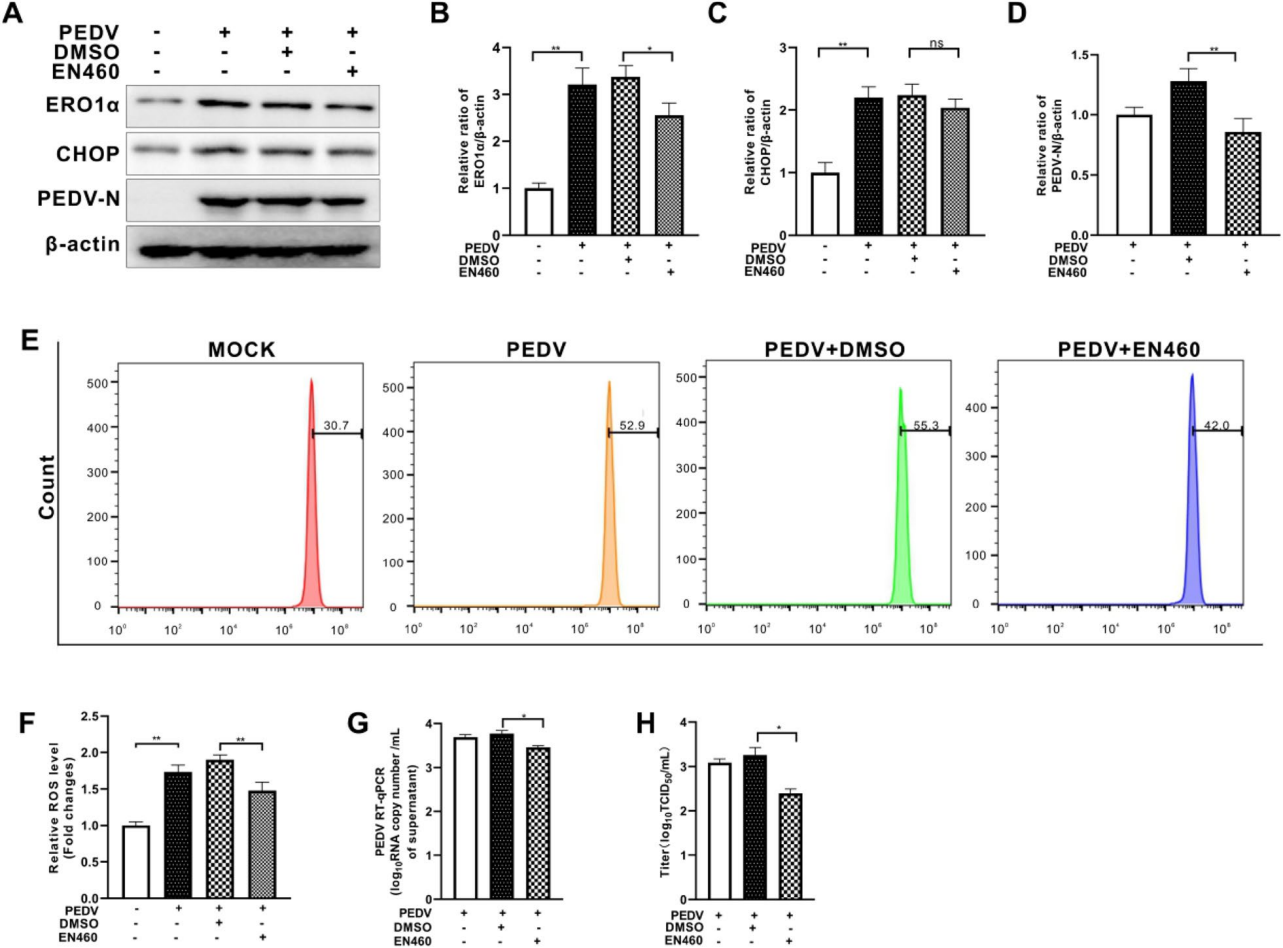
**Inhibition of ERO1α by EN460 reduced cytosolic ROS and viral replication in PEDV-infected Vero E6 cells.** Vero E6 cells were infected with PEDV (MOI = 0.001) with or without EN460 (5 μM). Dimethylsulfoxide (DMSO) was used as solvent control. **A** The effects of ERO1α inhibition on CHOP, ERO1α, and PEDV N expression were shown by Western blotting with β-actin used as loading control. **B** Ratios of ERO1α to β-actin. **C** Ratios of CHOP to β-actin. **D** Ratios of N to β-actin. **E** Cells collected at 36 hpi were measured for changes in cytosolic ROS level by flow cytometry after incubation with 10 μM DCFH-DA for 30 min at 37 °C. **F** The changes in cytosolic ROS levels were evaluated according to the mean value of DCFH-DA fluorescence intensity and expressed as fold changes between mock- and treated-cells. The effects of ERO1α inhibition by shRNA on viral replication, shown as viral RNA copy numbers measured by RT-qPCR (**G**) and virus titer (**H**). The bar charts in **B** to **D**, **F** to **H** represent mean ± SD of three independent experiments. ns, not significant; *, *P* < 0.05; **, *P* < 0.01.

ERO1α, a transcriptional target of CHOP, is closely related to endoplasmic reticulum protein loading and can induce ROS production [19]. To further elucidate the effect of ERO1α on ROS production and PEDV replication, we downregulated ERO1α expression by shRNA or by selective inhibitor EN460. We found that CHOP expression remained largely unchanged regardless of ERO1α knockdown in PEDV-infected cells (Figures 5A–C). However, generation of cytosolic ROS and viral replication, shown as N protein, viral RNA copy numbers, and virus titer, were diminished as a result of *ero1α*-silencing (Figures 5D–H). PEDV-infected cells treated with EN460 also resulted in reduced cytosolic ROS and viral replication, without affecting the expression of CHOP (Figure 6). These results indicate that PEDV infection enhanced ERO1α expression with perturbation of ER redox homeostasis.

### Scavenging of ROS with NAC decreased CHOP and ERO1α expression in PEDV-infected cells and repressed viral replication

**Figure 7:**
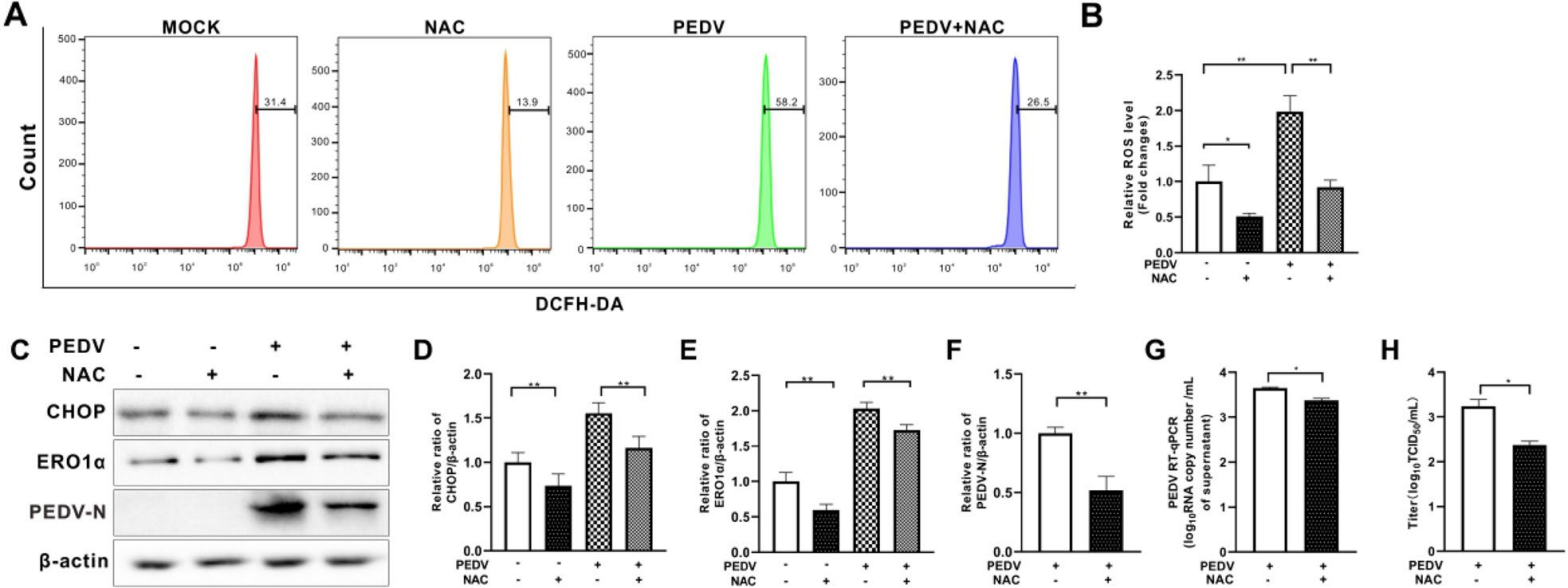
**Scavenging of ROS by N-acetyl-L-cysteine reduced CHOP and ERO1α expression, decreased cytosolic ROS, and limited viral replication in PEDV-infected Vero E6 cells.** Vero E6 cells were infected with PEDV (MOI = 0.001) in the presence or absence of *N*-acetyl-L-cysteine (NAC, 10 mM) for 36 h. **A** Cells collected at 36 hpi were measured for changes in cytosolic ROS level by flow cytometry after incubation with 10 μM DCFH-DA for 30 min at 37 °C. **B** The changes in cytosolic ROS levels were evaluated according to the mean value of DCFH-DA fluorescence intensity and expressed as fold changes between mock- and treated-cells. **C** The effects of ROS scavenging on CHOP, ERO1α, and PEDV N expression were shown by Western blotting with β-actin used as loading control. **D** Ratios of CHOP to β-actin. **E** Ratios of ERO1α to β-actin. **F** Ratios of N to β-actin. The effects of ROS inhibition on viral replication, shown as viral RNA copy numbers measured by RT-qPCR (**G**) and virus titer (**H**). The bar charts in **B**, **D** to **H** represent mean ± SD of three independent experiments. *, *P* < 0.05; **, *P* < 0.01.

We have shown that PEDV infection could induce ERO1α-mediated ROS. We next examined the role of ROS in PEDV replication. Antioxidant NAC was used to scavenge ROS in PEDV-infected Vero E6 cells (Figures 7A and B). We found that ROS removal restricted viral replication in Vero E6 cells, shown as reduced N expression, viral RNA copy numbers, and virus titer (Figures 7C, F–H). The CHOP and ERO1α levels were also reduced in NAC-treated PEDV-infected cells (Figures 7C–E).

## Discussion

Viruses regulate metabolism and survival of infected cells to assure their own reproduction and propagation. A hallmark of viral replication is the rapid modification of the host cell environment to facilitate the replication of the viral genome and the assembly of virus particles. For positive-strand RNA viruses, sites of viral RNA synthesis are composed of viral replicase proteins and host cell membranes, which together assemble into novel structures in the cytoplasm of infected cells. For example, picornavirus poliovirus generates rosette-like structures, flavivirus hepatitis C virus generates membranous webs, and coronaviruses generate double-membrane vesicles using membranes likely derived from the endoplasmic reticulum [3]. The massive production, folding, and modification of viral proteins pose a heavy burden to the ER. In addition, enveloped viruses must modify and perturb membranes to generate new virus particles, resulting in ER membrane depletion and morphological rearrangement. Often these modifications and perturbations trigger host cell stress responses, such as ER stress and UPR, which are generally engaged in coronavirus replication and in modulating the innate immune response of the host. A large amount of evidence indicates that coronavirus infection causes ER stress and triggers the UPR, thus regulating virus replication and proliferation [5, 6].

**Figure 8:**
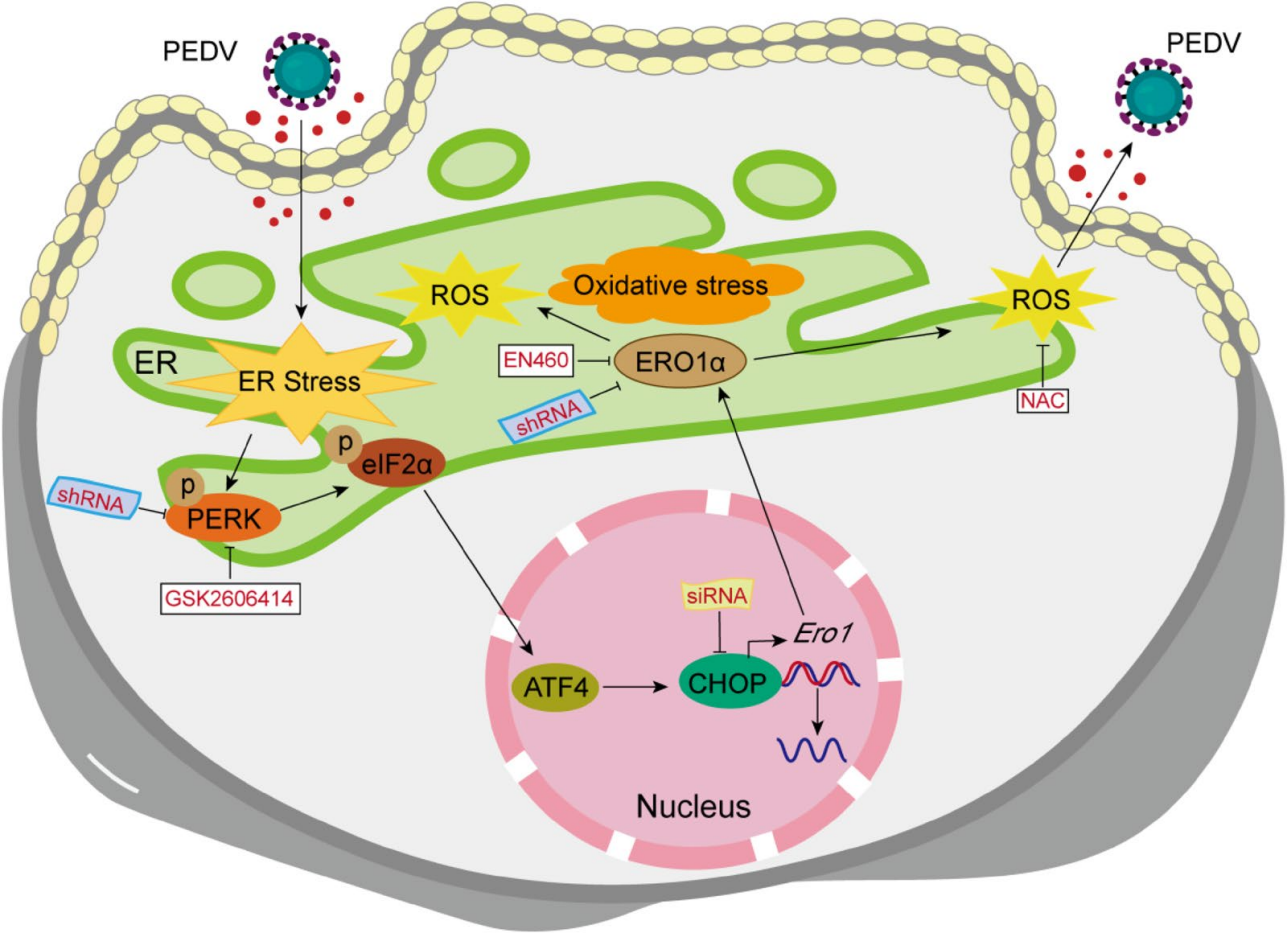
**Schematic illustration of PEDV-induced activation of the PERK-CHOP-ERO1α axis and increased generation of ROS to promote viral replication in Vero E6 cells.** PEDV infection induces ER stress by activating the PERK-eIF2α pathway which in turn activates CHOP. CHOP transcriptionally upregulates ERO1α which initiates oxidative stress by generating ROS during protein folding. Inhibition of PERK by shRNA or GSK2606414, CHOP knockdown by siRNA or ERO1α inhibition by shRNA or EN460 all lead to alleviation of ROS generation and reduced viral replication in PEDV-infected Vero E6 cells.

Our results clearly show that PEDV infection activated the PERK-eIF2α axis which, in turn, positively regulated viral replication via its downstream CHOP-ERO1α-ROS signaling, and that downregulation of PERK-eIF2α or inhibition of each of its downstream molecules suppressed PEDV replication (Figure 8). PERK-eIF2α is known to link with viral replication and pathogenesis [20]. Similar to our findings, inhibition of the PERK-eIF2α-ATF4 pathway was found to reduce mouse hepatitis virus (MHV) replication [21], and PERK inhibitors and its genetic depletion decreased MERS-CoV replication as well [22], revealing the importance of this pathway for successful coronavirus replication. Other viruses, such as porcine circovirus type 2, Newcastle disease virus, flavivirus Zika virus, etc., were also reported to utilize the PERK pathway to enhance their replication [23–26]. An earlier study showed that PERK knockdown or 4-phenylbutyric acid treatment increased PEDV propagation [27], while the underlying mechanism remains unknown. In addition, the PERK arm was found to negatively regulate replication of transmissible gastroenteritis virus and porcine hemagglutinating encephalomyelitis virus by suppressing protein translation [28, 29]. Thus, PERK-eIF2α signaling has different roles in infections of different viruses, and the detailed mechanisms need to be further examined. Besides PERK signaling, other UPR pathways were also implicated in virus replication. For example, a recent study found that cannabidiol inhibited SARS-CoV-2 replication in part by up-regulating the host IRE1α ribonuclease ER stress response and interferon signaling pathways [30].

The PERK/eIF2α pathway is known to induce CHOP, a transcriptional regulator of ERO1α [31, 32]. Oxidative maturation of secretory and membrane proteins in the ER is powered by ERO1 oxidases. Evidence suggests that oxidative protein folding is an important resource of ROS production in the cell [33]. Normally, ERO1α is responsible for disulfide bond formation by oxidizing protein disulfide isomerase [34]. This process is coupled with the production of hydrogen peroxide, suggesting that ERO1α has a strong association with protein load in the ER and can trigger ROS generation and contribute to ER-sourced oxidative stress [35]. We examined if increased expression of ERO1α and generation of ROS are associated with PERK activation by PEDV. This was achieved by inhibition of PERK via shRNA or GSK2606414, as well as by small interfering RNA-mediated knockdown of CHOP. Our results show that PEDV infection is capable of upregulating ERO1α expression and ROS generation through PERK activation. Pharmacological or genetic inhibition of UPR leads to significant reductions in titers of virions released from PEDV-infected cells. This corroborates the results seen in MHV- and SARS-CoV-2-infected cells [21].

We further investigated the role of ERO1α in PEDV-induced oxidative stress and viral replication by downregulating ERO1α expression by treating Vero E6 cells with shRNA or chemical inhibitor EN460. We found that inhibition of ERO1α suppressed ROS generation and viral replication. Induction of oxidative stress was first described in 1979 for the Sendai virus [36]. Since then, increasing studies have showed oxidative stress in various viral infections [37]. We further examined if viral replication could be affected by scavenging of ROS with NAC. The results indicate that NAC treatment of PEDV-infected cells led not only to the reduction of viral replication, but also to reduced expression of CHOP and ERO1α. Therefore, it is clear that PERK-induced activation of CHOP-ERO1α during PEDV infection contributes to excessive ROS generation of the stressed ER in favor of viral replication which could be counteracted by the antioxidant NAC. This is different from what has been observed for PEDV-induced autophagy where PERK and IRE1-mediated ER stress was presumably dependent on ROS from the mitochondria [12].

In conclusion, this study clearly shows that PERK-mediated ER stress was activated following PEDV infection, subsequently resulting in mobilization of its downstream molecules CHOP and ERO1α together with generation of excessive ROS. Because downregulation of each component of the PERK-CHOP-ERO1α-ROS nexus led to reduced viral replication, we propose that PEDV benefits its replication from inducing ER stress and oxidative stress, highlighting UPR as a promising antiviral target for adjunct therapy to combat infections by PEDV and possibly other coronaviruses as well.
